# Trophic Scaling and Occupancy Analysis Reveals a Lion Population Limited by Top-Down Anthropogenic Pressure in the Limpopo National Park, Mozambique

**DOI:** 10.1371/journal.pone.0099389

**Published:** 2014-06-10

**Authors:** Kristoffer T. Everatt, Leah Andresen, Michael J. Somers

**Affiliations:** 1 Centre for Wildlife Management, University of Pretoria, Pretoria, South Africa; 2 Centre for Invasion Biology, University of Pretoria, Pretoria, South Africa; Université de Sherbrooke, Canada

## Abstract

The African lion (*Panthera Leo*) has suffered drastic population and range declines over the last few decades and is listed by the IUCN as vulnerable to extinction. Conservation management requires reliable population estimates, however these data are lacking for many of the continent's remaining populations. It is possible to estimate lion abundance using a trophic scaling approach. However, such inferences assume that a predator population is subject only to bottom-up regulation, and are thus likely to produce biased estimates in systems experiencing top-down anthropogenic pressures. Here we provide baseline data on the status of lions in a developing National Park in Mozambique that is impacted by humans and livestock. We compare a direct density estimate with an estimate derived from trophic scaling. We then use replicated detection/non-detection surveys to estimate the proportion of area occupied by lions, and hierarchical ranking of covariates to provide inferences on the relative contribution of prey resources and anthropogenic factors influencing lion occurrence. The direct density estimate was less than 1/3 of the estimate derived from prey resources (0.99 lions/100 km^2^ *vs*. 3.05 lions/100 km^2^). The proportion of area occupied by lions was Ψ = 0.439 (SE = 0.121), or approximately 44% of a 2 400 km^2^ sample of potential habitat. Although lions were strongly predicted by a greater probability of encountering prey resources, the greatest contributing factor to lion occurrence was a strong negative association with settlements. Finally, our empirical abundance estimate is approximately 1/3 of a published abundance estimate derived from opinion surveys. Altogether, our results describe a lion population held below resource-based carrying capacity by anthropogenic factors and highlight the limitations of trophic scaling and opinion surveys for estimating predator populations exposed to anthropogenic pressures. Our study provides the first empirical quantification of a population that future change can be measured against.

## Introduction

The African lion (*Panthera Leo*), has suffered dramatic population and range declines over the last few decades and is currently listed by the IUCN as vulnerable to extinction [1]. Conservation management of the species requires reliable population estimates, however, these data are lacking for many of the continent's remaining populations; particularly those outside of protected areas that are exposed to human pressure [2], [3]. Quantifying the status of such populations is critical if we wish to promote the conservation of the species beyond a limited number of reserves [2].

As apex predators, lions are naturally limited by bottom-up prey resources and experience density dependence [4]. The relationship between predator biomass to prey biomass (averaged across all Carnivora) follows a ratio of 0.009/1 [5]. An association between lion density and lean prey density has been documented [6] and can be exploited to estimate lion density from prey density data [7]. However, demographic inferences based on trophic scaling assume that a predator population is subject only to bottom-up regulation, and are thus likely to produce biased estimates in systems with considerable top-down anthropogenic pressure [5], [8]. Lion populations in human influenced landscapes are susceptible to; persecution in defence of livestock [9], targeted poaching [10], by-catch of bushmeat hunting [11], over exploitation by trophy hunting [12] and disease transmitted from domestic animals [13]. The limiting effects of these top-down pressures may be felt by a population while being masked by intact prey resources [13], [14].

Comparing the observed differences between a realized density and potential density estimate based on estimates of prey biomass of an apex carnivore can provide evidence of non-density dependence, whereby variables other than resources are limiting a population [7]. Such comparisons are becoming increasingly important as Africa's rising human population exerts top-down pressures on predator populations both inside and outside protected areas [15], [16].

Here we investigate the status of lion in the developing Limpopo National Park (LNP) in Mozambique; a region where population data are lacking. LNP forms a component of one of Africa's Lion Conservation Units (Greater Limpopo LCU) and is contiguous with a protected population in the Kruger National Park (KNP) in South Africa [3], [17]. Unlike KNP, however, LNP is occupied by humans and livestock, and unregulated bushmeat hunting is not uncommon (*this study*). Prior to this study the only estimate of the lion population in LNP was derived from an opinion survey [18]. The use of opinion surveys can be inherently biased and produce overestimates of lion populations and should therefore be verified against empirical data [3]. The goal of this study was to provide empirical data on the status of lion in LNP, and to determine whether the population is limited by bottom-up prey resources or top-down anthropogenic factors. We compare a direct density estimate (realized density) obtained from a call-up survey [19] with an indirect density estimate obtained from trophic scaling (potential density) [7]. In addition, we employ replicated detection/non-detection surveys and an occupancy modelling technique that explicitly accounts for detectability [20] to estimate the proportion of area occupied by lion across a 2 400 km^2^ study area and to provide robust inferences on the factors limiting lion occurrence. We hypothesized that the lion population in LNP is currently limited by top-down anthropogenic pressures including agro-pastoralism and bushmeat poaching.

## Materials and Methods

### Ethics statement

We thank the Director of National Conservation Areas Mozambique for granting us the research permits (005-2011/003-2012) to conduct this study and Parque Nacional do Limpopo for supporting this research. All research methodologies used are considered non-invasive and so animal ethics approval was not required for this study.

### Study area and population

**Figure 1:**
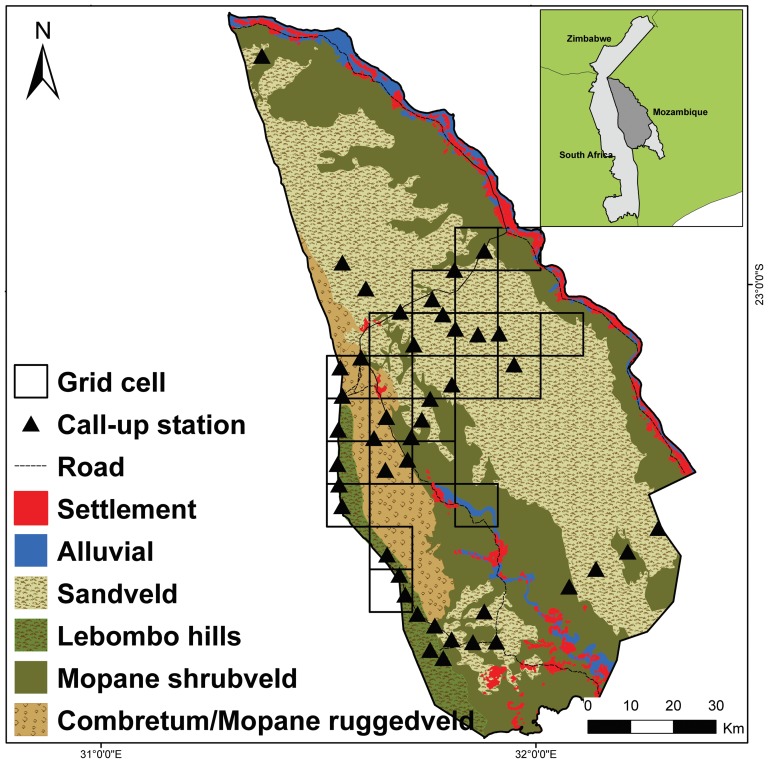
Survey effort in the Limpopo National Park (LNP), Mozambique. LNP is bounded to the west by the Kruger National Park in South Africa, characterized by formal protection and high wildlife densities, and to the east by the Limpopo River, characterized by agro-pastoralist settlements. Surveyed grid cells (100 km^2^) and call-up stations shown overlaid across a gradient of landscape types and human impact. Inset map: Location of LNP (dark grey) in relation to the Greater Limpopo Trans-frontier Park (light grey), including the region to the south of LNP which has been recently seperated by a wildlife barrier fence, and to Zimbabwe and South Africa.

This study was conducted in the LNP in south-western Mozambique, which forms a component of the Greater Limpopo LCU and of the Greater Limpopo Trans-frontier Park (GLTFP) with South Africa's KNP and Zimbabwe's Gonarezhou National Park. LNP is framed to the west by KNP, characterized by formal protection and high wildlife densities, and to the east, north-east and south by a near continuous band of agro-pastoralist settlements situated along the banks of the Limpopo River and Massingir Dam. There are additional smaller settlements situated along the Shingwedzi River that stretches north-south through the centre of the park. The human population within the central portions of LNP was estimated at 6 500 in 2003 with an additional 20 000 living in the eastern boundary settlements [21]. The cattle (*Bos primigenius*), population within LNP has been estimated at over 20 000 from 2010 aerial counts [22]. LNP officially includes 11 000 km^2^ (www.peaceparks.co.za), although excluding cultivated areas and a section to the extreme south that has been separated by a recently erected wildlife barrier fence, reduces the effective area of the park to 6 708 km^2^ (Fig. 1). There is limited infrastructure, including roads or tourist facilities. Mammalian fauna in Mozambique were largely decimated during 22 years of war (1964–1974; 1980–1992) [23]. Subsequent removal of portions of the South Africa-Mozambique border fence as part of the creation of the GLTFP (2000) has provided the opportunity for re-colonization of wildlife into LNP [24]. However, large mammal species' population recovery continues to be hindered by anthropogenic pressures including livestock husbandry, bushmeat poaching and poaching for elephant (*Loxodonta africana*) ivory and rhinoceros (*Ceratotherium simum*) horn (camera-trap data, *this study*). The IUCN [17] has identified the region as one of Africa's lion strongholds with an overall estimated population of 2 000, of which approximately 1 684 are in KNP [19]. An abundance estimate of 179 lions in LNP was derived from opinion based surveys [18]; however, prior to this study there had been no rigorous attempt to quantify the population.

The study area is comprised of woodland savannah plains with four distinct landscapes situated in approximate north-south orientation. These include; 1) sand plains characterized by low woodlands and thickets on deep sandy soils, the absence of well-defined drainage lines and the presence of ‘pans’ (seasonally flooded depressions), 2) combretum/mopane rugged veld characterized by tall shrublands and woodlands on clay soils, 3) mopane shrubveld characterized by thickets, short woodland and tall grasslands on calcareous soils, and 4) Lebombo hills characterized by short woodlands on undulating hills of stony, rhyolite soils [25]. The region receives an annual average 500 mm of rain, with the majority occurring between October and March [26].

### Survey design

#### Call-ups

To estimate lion density, a call-up survey was conducted during June and July 2012 *as per* Ferreira and Funston's [19] census of lions in KNP. Call-ups surveys employ a probability model to estimate lion abundance based on response counts to an auditory lure [27]. Demographically specific response probabilities, as well as a response radius needed to determine the effective area surveyed are estimated using calibration experiments [19]. Such calibration experiments were not possible in LNP due to low lion densities and insufficient road networks. We therefore assumed that the probabilities of lion response and response radius in LNP would be comparable to those in the adjoining and contiguous KNP.

To ensure the safety of the researchers when luring lions, we conducted call-ups from the back of a vehicle [19], which restricted our access to large portions of LNP that are not vehicle accessible. Given these constraints, we selected 43 call-up stations for sampling, located along all available roads, tracks and drivable routes. Although large portions of LNP were not accessible, the chosen call-up stations incorporated important environmental strata present in the park, including; 1) the most productive wildlife areas of the park (specifically areas of greater buffalo (*Syncerus caffer*) abundance, based on aerial survey data [22]), 2) a representative range of distances from human settlement areas, 3) a representative range of distances from the KNP boundary, and 4) major bio-physical features including the Limpopo River and distinguishing landscape types (Fig.1). Call-up stations were located a minimum of 5 km apart and sites were chosen to have relatively good visibility. In addition, call-up stations were located a minimum of 3 km from settlement boundaries or areas of high pastoralist use to avoid causing lion-human conflict.

#### Occupancy

We used an occupancy modelling approach that explicitly accounts for the probability of detection [20] to estimate the proportion of area occupied by lion and provide inferences on the ecological factors limiting their occurrence. Site occupancy models use replicated detection/non-detection surveys to estimate a detection probability (*p*) and derive unbiased estimates of species occurrence (*Ψ*). We make the following assumptions of an occupancy model for the estimator (*Ψ*) to be interpreted as the proportion of area occupied: 1) Sites are closed to changes in occupancy (i.e., are either occupied *by the species* or not for the survey duration; 2) Species are not falsely identified; 3) Detections are independent; and 4) Heterogeneity in occupancy or detection probability are modelled using covariates [20]. To estimate the proportion of area occupied by lion, sample units (sites) were defined as 10 km×10 km grid cells, which are comparable to estimated lion home ranges in the adjoining KNP (∼100 km^2^) [28]. We considered this size large enough to reduce spatial autocorrelation between sites, but conservative enough to assume that entire grid cells were occupied at sites where lions were detected (and thus reduce the chance of over-estimating the proportion of area occupied by lion). Our study design was constrained by lack of accessibility of large portions of LNP and the associated logistics of repeatedly accessing grid cells. Given these limitations, we selected 24 grid cells to be surveyed such that the resulting area followed a gradient of major bio-physical and anthropogenic features present in LNP (i.e., distinguishing landscapes, KNP boundary, drainage lines, and human settlements) and thus incorporated important strata (Fig. 1).

Lions are territorial felids, where males disperse from their natal range between the ages of 27–36 months [29]. To reduce the chance that a grid cell would become permanently vacated or colonized by the species over the survey period, we restricted our sampling duration to five months (May 7 to October 13, 2012). We employed two sampling methodologies; track surveys and camera-trapping. Sample occasions were represented by temporally replicated 3 km transects (replicates separated by more than 14 days) and 14 day camera-trap samples; considering this a reasonable amount of time to assume sample independence. Detections were represented by unambiguously identified lion tracks or photographs. Camera-traps and transects were located to maximize spatial representation of grid cells with a mean of two camera stations and two track transects per grid cell. To impose an order of randomness, each cell was divided into quadrants and one was randomly selected for obligate sampling. Due to logistical constraints, three cells were sampled in only one quadrant each, while the rest were sampled in two to four quadrants. Multiple surveys within the same quadrants were separated by more than 14 days. Of the 24 grid cells, 20 were sampled with camera-traps with a mean of 90 camera-trap nights per grid cell (range: 28–224 camera trap nights/grid cell) and 23 were sampled with track surveys with a mean of 13 kms walked per grid cell (range: 6–30 km/grid cell). Unequal sampling across sites is accounted for in the occupancy model [20].

We identified three predictor variables (covariates) that may explain lion occurrence in a human- influenced landscape, considering both bottom-up resources and top-down anthropogenic pressures. The covariates investigated were; preferred prey resources, bushmeat poaching and agro-pastoralist use (Table S1 in File S1). Considering that lions select home ranges based on characteristics that may change seasonally (i.e., buffalo or bushmeat poaching occurrence), we collected covariate data over the course of a year, from September 2011 to October 2012. To quantify the influence of preferred prey availability for lion we developed a probability of use model for buffalo; the most common preferred prey species of lion in the region [30], [31]. We make the assumptions of an occupancy model (as above), but note that the closure assumption could be relaxed because here we interpret our estimator (*Ψ*) as the *probability of site use* [32 pg. 105]. We developed the buffalo occupancy model based on replicated detection/non-detection surveys using camera-traps. Data were collected from 82 camera stations; each considered a buffalo sampling site. Buffalo sites were located to maximize spatial representation of lion grid cells with a mean of three buffalo sites per lion grid cell. Active camera stations were located more than 4 km apart. Sampling occasions were represented by 14 day camera-trap intervals.

Buffalo spatial use is influenced by the nutritional quality (nitrogen levels) of vegetation, water availability and predation risk [33]. To describe buffalo site use, we used six landscape covariates that account for variation in vegetation communities and underlying geology, surface water availability and anthropogenic disturbance. Covariates included; mopane shrubveld, sandveld, Lebombo hills, combretum/mopane rugged veld, distance to KNP boundary, distance to permanent water and distance to human settlements (Table S2 in File S1). Landscape covariates were extracted from a raster layer (www.peaceparks.co.za). All GIS analysis was done using the Spatial Analysis Toolbox in ArcGIS 9.3.1. (www.esri.com). The final mean buffalo occurrence covariate values were extracted for each of the 24 lion grid cells from a continuous (30 m resolution) Inverse Distance Weighted raster layer built from the weighted average occupancy estimates for each of the 82 buffalo sites. We assumed that our buffalo occupancy model is representative of a preferred prey encounter probability for lion.

We used a similar approach to quantify the impact of bushmeat poaching on lion occurrence. A bushmeat poaching occupancy model was built from photographic data of humans carrying snares, traps, spears or bows, domestic hunting dogs (*Canis lupus familiaris*), and mammals with snares around their necks or with obvious snare wounds. Data were collected from 82 camera stations (as above); each considered a bushmeat poaching sampling site, with a mean of three bushmeat sites per lion grid cell. Sampling occasions were represented by 14 day camera-trap intervals. We make the assumptions of an occupancy model (as above), but again note that the closure assumption could be relaxed because we interpret our estimator (*Ψ*) as the *probability of site use*.

We identified six covariates that could account for heterogeneity in bushmeat poaching site use based on optimal foraging theory; considering risk, effort and reward to hunters [34], [35]. Covariates included; ranger patrols, distance from villages, distance from tracks/trails, proximity to waterholes and rivers, the relative abundance of bushmeat and the relative biomass of bushmeat (Table S3 in File S1). We considered ‘bushmeat’ species that were observed in snares over the course of this study including; buffalo, impala (*Aepyceros melampus*), greater kudu (*Tragelaphus strepsiceros*) and nyala (*Tragelaphus angasii*). Site specific relative abundance of bushmeat was estimated from a continuous raster layer built from raw data (237 points) from the most recently available (2010) fixed-wing aerial survey. The aerial survey used a total area count strip-transect method, sampling every third transect [22]. Relative bushmeat biomass was measured as the relative abundance of each species multiplied by ¾ average female weights of the species [7]. During the survey period, patrol effort in LNP was limited and primarily restricted to monthly patrols of the main roads (park management *pers. com*). Considering that bushmeat poachers may avoid these areas, we used proximity to main roads as a proxy for patrol effort. Proximity to tracks/trails, main roads, rivers, and settlements were measured from a landscape raster (www.peaceparks.co.za) using the Spatial Analysis tool in ArcGIS 9.3.1. Considering that the cameras were disguised and used infra-red flashes (see below), we could think of few covariates to explain heterogeneity in detection. We experienced 10 camera thefts over the course of the study, primarily along tracks (*vs*. natural landscape features), and therefore considered that tracks may influence detectability. The final mean bushmeat poaching occurrence covariate values were extracted for each of the 24 lion grid cells from a continuous (30 m resolution) Inverse Distance Weighted raster layer built from the weighted average occupancy estimates for each of the 82 bushmeat poaching sites. We assume that our occurrence probability model for bushmeat poaching is representative of an encounter probability for lion. To quantify the impact of agro-pastoralism on lion occurrence, we considered the mean Euclidean distance (from each 30 m pixel in a grid cell) to a settlement boundary. We accounted for heterogeneity in lion detectability between survey methodologies using a survey-specific covariate. We did not attempt to model differences in detectability between camera brands in any of our occupancy models, considering trigger speed and detection zones between camera brands comparable (details below).

### Data collection

#### Call-ups

At each station, a four minute recording of a buffalo calf distress call was broadcast twice followed by two minutes of silence for a total period of 60 min. The call was recorded onto a SD card and broadcast thru a 12 volt 100 watt amplifier (Stewart PA100-MP3, Sonora, USA), powered by the vehicle's battery, and two 40 Watt horn speakers with driver units (Show TC-40P, Kyung Gi-Do, Korea). The call was broadcast at full volume from the speakers mounted 180° from each other, 3 m off of the ground on a steel tripod placed 20 m from the vehicle. The speakers were rotated 90° one time after 30 min to provide 360° coverage. We scanned for eye shine at three to five min intervals using a spotlight (Lightforce SL240 Blitz, Hindmarsh, Australia) with a red filter, and listened for animal movements during the periods of silence. We recorded the number of adult and sub-adult lions and the presence or absence of cubs [19].

#### Camera-traps

To maximize the probability of detecting lions, camera stations were deployed at waterholes and on dirt tracks, game trails, and river edges used for travel by carnivores. Digital motion-activated cameras with infra-red flashes were used (15 Reconyx HC500 (Wisconsin, USA) (trigger time of 0.97 s, detection zone approximately 24 m), 7 Spy Point Tiny-W2 (Québec, Canada) (trigger time of 0.91 s, detection zone approximately 17 m), 10 Bushnell Trophy Cam (Beijing, China) (trigger time of 0.66 s, detection zone approximately 18 m) (http://www.trailcampro.com/trailcamerareviews.aspx)). Risk of theft and vandalism required substantial effort to conceal the cameras. Each camera was enclosed in a steel box, secured using cable locks and camouflaged with vegetation. Vegetation that could falsely trigger the cameras was removed with care to reduce human attention to the site.

#### Track transects

Track transects were conducted on foot due to the lack of road networks. Track transects followed a main path of travel, (i.e., track, trail or river edge), and were conducted by KE and LA in early morning or late afternoon hours where substrate was adequate for tracking. The detection or non-detection of lion tracks was recorded for each 3 km transect sample.

#### Minimum number alive and mortalities

We determined the minimum number of individual lions alive (with identification based on sex, age and distinguishing scars) and recorded the minimum number of lion mortalities within the study area (i.e., the area encompassed by the 24 grid cells and call-up stations; Fig. 1)

### Analytical methods

#### Call-ups

Lion abundance was estimated from call-up data using a probabilistic approach first developed by Mills et al. [27] for spotted hyenas (*Crocuta crocuta*) and refined for lions by Ferreira and Funston [19]. Probabilities and response radius were borrowed from Ferreira and Funston's [19] calibration experiments in KNP; each station was assumed to have sampled an area of 57.7 km^2^.

#### Trophic scaling

To estimate the ecological carrying capacity of lion in LNP, we used Hayward et al.'s [7] regression model relating lion density to biomass of preferred prey species. Prey biomass was calculated using ¾ of the adult female weight [7] of each species considered preferred prey by lions [31] and available in LNP, including; buffalo, blue wildebeest (*Connochaetes taurinus*), giraffe (*Giraffa camelopardalis*) and plains zebra (*Equus burchelli*), multiplied by species minimum counts obtained from 2010 aerial survey of LNP [22].

#### Occupancy models

Site occupancy (*Ψ*) and probability of detection (*p*) were estimated using maximum likelihood functions [32] and the single season option in the program PRESENCE Version 5.5 [36]. Continuous site covariates were standardized on a z-scale. We tested for collinearity between variables using a cut-off of *r* = 0.5. Models were ranked based on Akaike Information Criterion (AIC), using AICc adjusted for small sample size, with the sample size set at the number of sampling sites. Models with a ΔAICc<2 were considered to be strongly supported. We considered a candidate set of all models ΔAICc<7 whose combined weights ≥0.95 (i.e., 95% confidence set), excluding models that did not reach numerical convergence. AICc weights were used to determine the weight of evidence for each model, and were summed for each covariate in the 95% confidence set [37]. Variables with high summed model weights were considered more important in explaining heterogeneity in occupancy. The direction of influence of covariates was determined by the sign of the β-coefficients [32]. Covariates were considered to have strong or robust impact if β±1.96 x SE did not include zero. A weighted model averaging technique was used to calculate overall estimates of 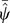 and 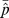 [38]. A goodness of fit test using 10, 000 bootstrap samples and a Pearson's chi-squared statistic was performed on the most saturated model [38].

#### Buffalo occupancy model

A detection/non-detection matrix was constructed for each of 82 buffalo sites, recording a ‘1’ or ‘0’ where buffalo were detected or not, respectively. The covariates ‘combretum/mopane rugged veld’ and ‘sand plains’ were found to be correlated (r = −0.5), as were ‘KNP’ and ‘sand plains’ (r = 0.7) and ‘KNP’ and ‘Lebombo hills’ (r = −0.6) and were not included in the same models. To determine the factors that best describe buffalo occurrence, we compared all possible (non-correlated) combinations of occupancy covariates (60 models).

#### Bushmeat poaching occupancy model

Following the same procedure as above, a detection/non-detection matrix was constructed for each of 82 bushmeat poaching sites, recording a ‘1’ or ‘0’ where bushmeat poaching was detected or not, respectively. The covariates ‘ranger patrol’ and ‘settlement’ were found to be correlated (r = 0.7) and were not included in the same models. First, we evaluated the covariate ‘track’ to describe heterogeneity in bushmeat hunting detection probability. We included the covariate for ‘track’ in all subsequent analysis; this model was strongly supported and ranked higher than the model that assumed detectability was constant (ΔAICc = 20.44). To determine the factors that best describe bushmeat poaching occurrence, we compared all possible (non-correlated) combinations of occupancy covariates (47 models).

#### Lion occupancy model

A detection/non-detection matrix was constructed for each of 24 lion grid cells, recording a ‘1’ or ‘0’ where lion were detected or not, respectively. Following this, a survey-specific matrix was constructed to account for differences between the two sampling methods, recording a ‘1’ for cameras and a ‘0’ for tracks. To determine whether top-down anthropogenic factors or bottom-up prey resources were limiting the lion population in LNP, we compared a simple set of three univariate models to the model that accounts for variation in lion detection with survey method.

http://dx.doi.org/10.6084/m9.figshare.931785

http://dx.doi.org/10.6084/m9.figshare.928555

## Results

We recorded a minimum of 34 lions in the study area between September 2011 and November 2012. These included 22 individuals identified from the camera trapping survey, four identified only from the call-up survey, six that we opportunistically observed and an additional two that were photographed by a park contractor. The overall sex ratio was 0.9 females to 1.0 male. We recorded five lion mortalities, all human-caused, in the study area during September 2011 to November 2012.

### Call-ups density estimate

**Figure 2:**
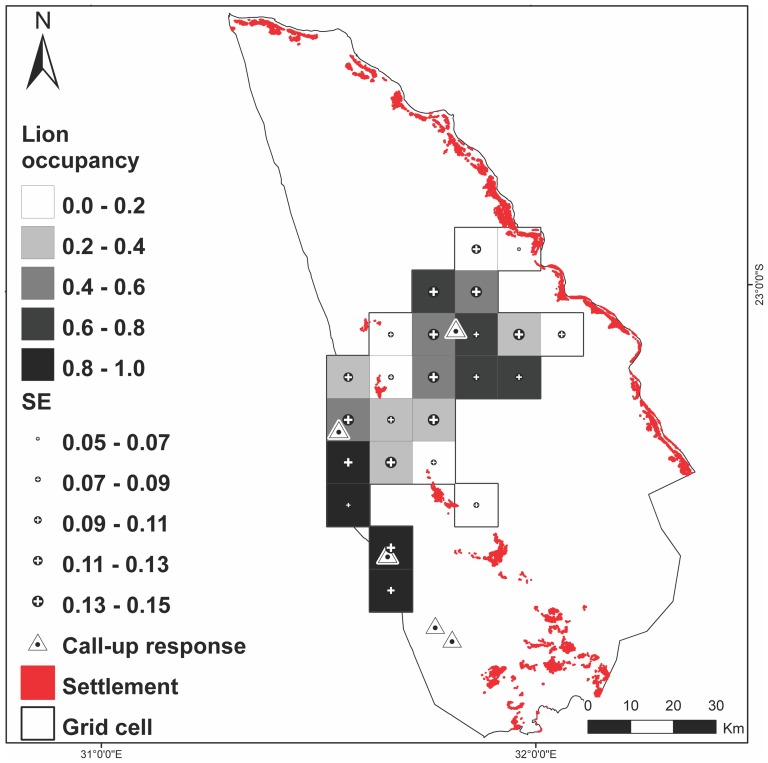
Spatial distribution of lion site occupancy and locations of call-up detections in the Limpopo National Park, Mozambique. Occupancy estimates are based on the averaged model (∑w>0.95) from 206 (mean = 9/grid cell) surveys of 24 (100 km^2^) grid cells. Call-up detections are from a total of 43 stations.

We recorded 13 lion responses at five of the 43 call-up stations, providing a mean of 0.27 lions per sample (Fig. 2). Lions were easily distinguished from sympatric species (i.e. spotted hyena and leopard, *Panthera pardus*), and lion eye shine was readily detectable, including through relatively thick vegetation. We estimated the effective area surveyed to include 1 852 km^2^, which represents approximately 28% of the potential lion habitat in LNP (calculated using published response radius [19] and excluding a 2 km buffer around cultivated areas). Respondents included five adult males, seven adult females and one cub. Two of the responding groups of lions (3 x adult females and 3 x adult females) were counted at adjoining stations on consecutive nights. Ferreira and Funston [19] attempted to account for possible bias caused by double counting lions by developing a probability of repeat response; however in five trials they did not record any repeat responses. We attempted to account for bias induced by the possibility of double sampling the three lionesses by calculating abundance both with and without the second group and taking the average of the two. This provided an abundance estimate of 66.2 and an overall density estimate of 0.99 lions/100 km^2^ in LNP (excluding the areas covered by a 2 km buffer around cultivation). We were unable to calculate variance for these estimates.

### Indirect density estimate

Aerial count data of 475 points of preferred prey [22] produced an average available biomass estimate of 50.07 kg/km^2^. Trophic scaling of the available biomass produced a density estimate of 3.05 lions per 100 km^2^. This estimate is more than three times greater than that produced from the call-up survey.

### Buffalo site use

Buffalo were detected on 105 sampling occasions (collapsed from 1 264 independent photo events). The final data set consisted of 369 sampling occasions, with a mean of five sampling occasions per buffalo site. The weighted average probability of detecting buffalo where they occurred on a single survey was 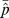 = 0.368 (SE = 0.041). The summary of model selection procedure is provided in Table S4 in File S1. Buffalo site use was considerably higher closer to the KNP border and further from settlements, and considerably lower in the mopane shrubveld. Buffalo site use was also generally higher in closer proximity to water and lower in the combretum/mopane rugged veld (Table 1). Site level occupancy estimates ranged from 0.008 to 0.887 with a weighted average of 0.416 (SE = 0.084). There was no evidence lack of fit (p = 0.09) or over-dispersion (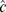 = 1.43).

**Table 1:** β- coefficient estimates for covariates influencing buffalo site use (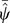) in order of their summed model weights (∑w).

Occupancy Covariate	∑ w (%)	*β* coefficient	SE
KNP	80.1	1.36*	0.47
Settlement	50.8	−1.05*	0.51
Mopane shrubveld	49.4	−2.16*	1.09
Combretum/mopane rugged veld	41.6	−1.28	0.73
Water	13.1	0.28	0.33

^*^ Indicates covariate has robust impact (*â*±1.96 x SE not overlappling 0).

### Bushmeat poaching site use

Camera-traps recorded 89 events of humans carrying bows, snares, traps or spears, 66 domestic hunting dog events and 21 events of mammals carrying snares or with snare wounds. These data were collapsed into 47 bushmeat poaching detections. The final data set consisted of 375 sampling occasions, with mean of five sampling occasions per bushmeat sampling site. Model averaged estimates showed that the probability of detecting bushmeat poaching at a site where it occurs was low (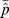 = 0.165, SE = 0.027) (Table S5 in File S1). Site level occupancy estimates ranged from 0.000 to 0.994 with a weighted average of 0.799 (SE = 0.050).

Bushmeat poaching site use increased strongly with the relative abundance of bushmeat but decreased with the relative biomass of bushmeat (Table 2). These results indicate use of sites with a relatively higher abundance of the smaller-bodied antelopes that we considered (i.e., impala). Bushmeat poaching site use was also considerably higher closer to tracks/trails and settlements and lower along the main road. There was no evidence lack of fit (p = 0.79) or over-dispersion (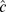 = 0.44).

**Table 2:** β- coefficient estimates for covariates influencing bushmeat poaching site use (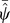) in order of their summed model weights (∑w).

Occupancy Covariate	∑ w (%)	*β* coefficient	SE
Bushmeat abundance	99.4	429.632*	3.588
Bushmeat biomass	99.4	−134.160*	3.493
Settlement	72.5	16.460*	3.559
Tracks	38.0	15.250*	6.502
Ranger patrol	5.0	−0.348	0.724

^*^ Indicates covariate has robust impact (*â*±1.96 x SE not overlapping 0).

### Lion occupancy

A total camera-trapping effort of 1 845 camera-trap nights resulted in 26 lion detection events (collapsed from 218 photographs of lions) from 10 of 38 camera stations in seven grid cells. A total of 303 km of track surveys were walked, resulting in 33 lion track events in nine grid cells. The final data set consisted of 206 sampling occasions with a mean of nine sampling occasions per lion grid cell. The weighted average probability of detecting lions where they occurred on a single survey was relatively high; 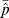 = 0.274 (SE = 0.066). The weighted average estimate of the proportion of area occupied by lion was 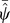 = 0.439 (SE = 0.121) (Table 3), or lions occupied approximately 44% of the 2 400 km^2^ survey area. The spatial distribution of lion occurrence in the study area is provided in Fig. 2.

**Table 3:** Model selection procedure for factors influencing lion occupancy (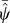) across 24 (100km^2^) sites in the Limpopo National Park, Mozambique.

Models	ΔAICc	*w*	K	*−*2l	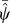	(±SE)	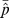	(±SE)	*β*-coefficient	SE
Ψ(V)p(M)	0.00	0.627	4	129.97	0.441	0.119	0.274	0.066	−2.02*	0.93
Ψ(P)p(M)	1.27	0.332	4	131.24	0.433	0.125	0.276	0.065	6.59*	2.93
Ψ(.)p(M)	6.23	0.028	3	139.11	0.458	0.120	0.268	0.066		
Ψ(B)p(M)	7.79	0.013	4	137.76	0.462	0.167	0.267	0.066	−2.92	2.58
**Model Average**					0.439	0.121	0.274	0.066		

Â-coefficient estimates for covariates strength and direction of influence are also shown.

Covariates considered include; settlement (V), buffalo (preferred prey) (P) and bushmeat poaching (B). Detectability (*p*) varies with method (M). Estimates of 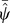 and 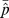
and associated standard errors (SE). Ø(.) assumes lion occupancy is constant, ΔAICc is the difference in AICc values between each model with the low AICc model, *w* is the AICc model weight, K is the number of parameters in the model, and −2l is twice the negative log-likelihood.

* Indicates covariate has robust impact (â±1.96 x SE not overlapping 0).

In agreement with our hypothesis, there was evidence that lions are limited by anthropogenic pressure in LNP. The greatest contributing factor (*w* = 63%) to lion occurrence was a strong negative association with agro-pastoralist settlements; (β = −2.02, SE = 0.93) (Table 3). Mean site estimates were 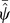 = 0.182 (SE = 0.098) at sites less than 10 km from settlements (10 sites) and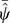 = 0.591 (SE = 0.129) at sites equal to or greater than 10 km from settlements (14 sites). There was also support for the hypothesis that lions were limited by prey resources (*w* = 33%). Lions were strongly positively associated (β = 6.59, SE = 2.93) with sites where they had a greater probability of encountering buffalo (Table 3). Mean site estimates were 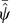 = 0.609 (SE = 0.124) at sites with greater than 50% buffalo occupancy (five sites) and 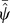 = 0.343 (SE = 0.113) at sites with less than 50% buffalo occupancy (19 sites). We found no support for the hypothesis that lions were limited by bushmeat poaching at the spatial scale examined (ΔAICc = 7.79), however, lions did tend to occur less at sites with a greater probability of encountering bushmeat poaching (Table 3). There was no evidence of lack of fit (p = 0.52) or over-dispersion (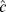 = 0.49).

## Discussion

### The influence of prey, bushmeat poaching and pastoralism on lions

In agreement with our hypothesis, our results indicate that the lion population in LNP is limited by top-down anthropogenic pressures. Comparing our direct density estimate with the estimate obtained from trophic scaling indicates that the lion population in LNP is currently at less than 1/3 of its carrying capacity based on prey resources. As an apex predator, lions are naturally limited by bottom-up prey resources [4]–[7] and therefore the observed disparity between realized and potential densities suggests the influence of external top-down, anthropogenic pressures. Additionally, during the survey we documented five lions snared or shot by bushmeat poachers or pastoralists. The hypothesis of top-down anthropogenic pressures limiting the lion population in LNP is further supported by the observed relationships between lion occupancy and the explanatory covariates investigated.

In agreement with known species relationships [31], there was strong support for the hypothesis that lions were bottom-up limited by prey resources. Nevertheless, there was slightly more support for the top-down limiting hypothesis; the greatest predictor of lion occurrence in LNP was a strong negative correlation with agro-pastoralist settlements (Table 3). Persecution by farmers and pastoralists has contributed considerably to the decline of lion populations and the reduction of lion range across Africa [3], [17], [39] and it is therefore not surprising that the pastoralism covariate carried the greatest weight in explaining lion occurrence in a region impacted by humans and livestock. We estimate that lions occupy only approximately 44% of our 2 400 km^2^ sample area in LNP. The distribution of their occurrence suggests that lions may be suffering from persecution around agro-pastoralist settlement areas and/or are exhibiting spatial avoidance of these activities (Fig. 2). Interestingly, our analysis indicated that lion occurrence was not significantly influenced by bushmeat poaching activities. We caution, however, against the interpretation that lion populations are not limited by the pressures of bushmeat poaching. In order to estimate the proportion of area occupied by lion, we examined the influence of variables on lion occurrence at the home-range spatial scale only. While bushmeat poaching did not appear to influence lion occurrence at this scale, the same relationship may not hold at smaller spatial scales [40]. We suggest that further research should consider the influence of scale when investigating the limiting effects of anthropogenic pressures on lions. It is also important to note that while the level of bushmeat poaching present in LNP may not influence the probability that a home range-sized sample unit is occupied by the species, it may, however, influence the local abundance of lion.

### Determining abundance of lions in a human-impacted landscape

Our study provides the first empirical data on a lion population exposed to anthropogenic pressures in a developing National Park in Mozambique. Prior to this study, the only population estimate available for lions in LNP was derived from an expert opinion survey, which produced an estimate of 179 individuals [18]. Our results suggest that the actual number of lions in the park is approximately one third of their estimate. Our estimate of 66 lions in LNP excludes the region south of the wildlife barrier fence (Fig. 1), and is based on the assumption that areas within 2 km of cultivation cannot be considered suitable habitat for lions. If we applied our call-up density estimate of 0.99 lions per 100 km^2^ to the full 11 000 km^2^ area of the park without consideration to human disturbance, than our abundance estimate for LNP would increase to 108 lions. However, we feel that this would be a gross overestimate of the actual population. Our call-up survey sampled approximately 28% of the available lion habitat in LNP, however, we do acknowledge the possible bias in extrapolating our density estimate across areas of the park that were not sampled due to lack of vehicle accessibility (Fig. 1). We attempted to account for variability that may arise in lion densities by sampling from important environmental strata including the full range of distances from human settlements and the KNP boundary as well as distinguishing landscape types. It is still possible that we may be underestimating lion densities if areas inaccessible by vehicle have lower human impact (i.e., lower cattle grazing and bushmeat poaching) and therefore higher lion densities. However, neither cattle grazing nor bushmeat poaching are road dependent in LNP; both activities are conducted by people that walk long distances using trail networks (camera-trap data, *this study*). Therefore, based on our knowledge of the park, we believe that the distance from a road should be of less consequence to the effects of these anthropogenic factors on lion density than is the distance from a human settlement. By sampling across a representative range of distances from human settlements in LNP we reason that we were able to account for variation in lion density that may arise from variation in human pressures. A further consideration is that the 2010 aerial surveys reported relatively low ungulate abundance, including low buffalo abundance, in the two large un-sampled areas in the park. The majority of buffalo were found along the unpopulated stretches of the Shingwedzi River valley and close to the KNP border [22]; areas that we were able to include in our sample. It is therefore unlikely that lion density in either of the un-sampled areas would be significantly higher than the average density for the areas that we were able to sample. Despite the limitations of our study design, our estimate comprises the only empirical population data on lions in LNP and thus is the most reliable estimate available. In light of the overall lack of empirical data on lion populations in this region and across much of Africa [3] and the declining conservation status of the species [3], [41] we believe that our initial estimates are a valuable contribution to the conservation management of lions in the region.

A possible bias in our trophic scaling estimate could have arisen because it was based on aerial prey data obtained in 2010 [22] and prey populations may have since changed. However, the competing forces of bushmeat poaching activities reducing ungulate populations and natural immigration from KNP augmenting ungulate populations should dampen these changes.

Density estimates based on trophic scaling assume that a predator population is subject only to bottom-up regulation. With increasing human disturbance, simple bottom-up regulatory systems are likely becoming increasingly rare across Africa and much of the world [42], [43]. While estimating lion densities using trophic scaling may be a practical means of acquiring empirical population data, the failure to account for top-down anthropogenic pressure can result in overestimations of predator populations. Such overestimates can lead to erroneous status assessments and populations going overlooked that are in need of conservation attention. A trophic scaling approach for estimating lion abundances may therefore have limited usefulness in human-impacted systems [5].

### Management implications

Our results indicate that the lion population in LNP is currently held below carrying capacity by anthropogenic factors. That lions were strongly negatively associated with settlement areas suggests that lions may be suffering mortality due to persecution and/or spatially avoiding these sites (Fig. 2). Furthermore, a negative association with settlements along the Limpopo River may be indicative of edge effects [15]. The long term development plan for LNP includes the re-settlement of the central settlements to areas along the Limpopo River [44]. Reduction of human-impact in the core of the park may permit the lion population to increase towards a prey-based carrying capacity and increase their proportion of area occupied. However, increasing human density along the Limpopo River may decrease landscape permeability for lions between the Kruger-Limpopo system and other areas of the Greater Limpopo LCU (i.e., Gonarezhou National Park in Zimbabwe and Banhine and Zinave National Parks in Mozambique), thus compromising the viability of a potential meta-population.

Altogether, our results have important conservation implications when placed in context of the Greater Limpopo LCU. We expect that both the population and range estimates of Chardonnet et al. [18], IUCN [17] and Riggio et al. [3] for the Mozambican component are unrealistically optimistic and that the lion population is likely highly fragmented and requires conservation interventions. We suggest that landscape-scale, spatially replicated occupancy surveys [45] could be extended across the Greater Limpopo LCU to identify sub-populations, potential corridors and limiting factors, which if coupled with demographic data could be used to assess the management actions required to maintain a viable lion meta-population [46], [47].

## Supporting Information

File S1 **This file contains Table S1 to S5.** Table S1. Covariates expected to influence occurrence of lion. Table S2. Covariates expected to influence occurrence of buffalo. Table S3. Covariates expected to influence occurrence of bushmeat poaching. Table S4. Summary of model selection procedure for factors influencing buffalo site use (*Ψ*) across 82 sites in the Limpopo National Park, Mozambique. Table S5. Summary of model selection procedure for factors influencing bushmeat poaching site use (*Ψ*) across 82 sites in the Limpopo National Park, Mozambique.
(DOCX)
