# Supplementary material for: Trophic Scaling and Occupancy Analysis Reveals a Lion Population Limited by Top-Down Anthropogenic Pressure in the Limpopo National Park, Mozambique
Source: PLoS One. 2014 Jun 10;9(6):e99389. doi: 10.1371/journal.pone.0099389 (PMC4051697; doi:10.1371/journal.pone.0099389)
Supplement: File S1 — This file contains Table S1 to S5. Table S1. Covariates expected to influence occurrence of lion. Table S2. Covariates expected to influence occurrence of buffalo. Table S3. Covariates expected to influence occurrence of bushmeat poaching. Table S4. Summary of model selection procedure for factors influencing buffalo site use (Ψ) across 82 sites in the Limpopo National Park, Mozambique. Table S5. Summary of model selection procedure for factors influencing bushmeat poaching site use (Ψ) across 82 sites in the Limpopo National Park, Mozambique. (DOCX) [file pone.0099389.s001.docx]

**Supplementary Information**

**Table S1.** Covariates expected to influence the proportion of area occupied by lion.

| **Covariate** | **Relationship to lion fitness** | **Description** | **Sampling range** |
| --- | --- | --- | --- |
| Buffalo | Availability of | Probability of buffalo site | - 1. – 0.6 |
|  | preferred prey | use (encounter probability) | mean = 0.4 |
| Bushmeat | Targeted or accidental | Probability of bushmeat poaching | - 1. – 1.0 |
| poaching | snaring | site use (encounter probability) | mean = 0.6 |
| Settlement | Persecution in defense | Proximity to agro-pastoralist | 2.0 - 20.8 |
|  | of livestock | settlements (km) | mean = 11.1 |

**Table S2.** Covariates expected to influence buffalo site use.

| **Covariate** | **Relationship to** | **Description** | **Number of sites/** |
| --- | --- | --- | --- |
|  | **Buffalo fitness** |  | **Sampling range** |
| KNP | Protected population | Proximity to KNP border (km) | 0.0 - 50.0 |
|  |  |  | mean = 21.9 |
| Mopane shrubveld | Nutritional variation | Shrublands and thickets | n = 11 |
|  |  | on calcerous soil |  |
| Combretum/mopane | Nutritional variation | Woodlands and shrublands of | n = 17 |
| rugged veld |  | on clay soils |  |
| Lebombo hills | Nutritional variation | Short woodlands and shrublands | n = 11 |
|  |  | on rhylolite soils |  |
| Sand plains | Nutritional variation | Short woodlands and thickets | n = 41 |
|  |  | on sandy soils |  |
| Water | Water availability | Proportion of site (50 m buffer) | 0.0 -1.0 |
|  | Nutritional variation | overlapping a drainage line | mean = 0.1 |
| Settlement | Direct persecution and | Proximity to agro-pastoralist | 0.5 – 22.7 |
|  | competion with cattle | settlement (km) | mean = 11.7 |

**Table S3.** Covariates expected to influence bushmeat poaching site use.

| **Covariate** | **Relationship to** | **Description** | **Sampling range** |
| --- | --- | --- | --- |
|  | **bushmeat poaching** |  |  |
| Bushmeat | Availability of | Relative abundances of bushmeat | 1.2 – 8.4 |
| abundance | bushmeat | obtained from aireal | mean = 3.6 |
|  |  | count data (23) including: |  |
|  |  | Impala, *Aepyceros melampus* |  |
|  |  | Kudu, *Tragelaphus strepsiceros* |  |
|  |  | Nyala, *Tragelaphus angasii* |  |
|  |  | Waterbuck, *Kobus ellipsiprymnus* |  |
|  |  | Buffalo, *Sycerus caffer* |  |
| Bushmeat | Profitability of bushmeat | Relative biomass (kg/site) of above | 70 – 3 079 |
| biomass |  | bushmeat points multiplied by ¾ adult | mean = 437 |
|  |  | female weight (7) |  |
| Track | Ease of access | Proximity (km) to tracks/trails | 0.0 – 3.4 |
|  |  | measured in ArcGIS | mean = 0.3 |
| Water | Increased probabilty | Proximity (km) to rivers measured in | 0.0 – 9.3 |
|  | of encountering bushmeat | ArcGIS | mean = 3.7 |
| Settlement | Proximity decreases | Proximity (km) to settlements | 0.5 – 22.7 |
|  | energetic costs of meat | measured in ArcGIS | mean = 11.7 |
|  | retrieval |  |  |
| Ranger | Risk of arrest, fines | Proximity (km) to road measured in | 0.0 – 22.9 |
| patrol | or dogs destroyed | ArcGIS | mean = 7.7 |

**Table S4.** Summary of model selection procedure for factors influencing buffalo site use (Ψ) across 82 sites in the Limpopo National Park, Mozambique.

| **Models** | **∆AICc** | ***w*** | **K** | ***-*2l** |
| --- | --- | --- | --- | --- |
| Ψ(K+M+C)p(.) | 0.00 | 0.134 | 5 | 264.43 |
| Ψ(K+V)p(.) | 0.03 | 0.132 | 4 | 266.73 |
| Ψ(K+V+C)p(.) | 0.32 | 0.114 | 5 | 264.75 |
| Ψ(K+V+M)p(.) | 0.54 | 0.102 | 5 | 264.97 |
| Ψ(K+M+C+V)p(.) | 0.62 | 0.098 | 6 | 262.72 |
| Ψ(K+M)p(.) | 0.79 | 0.090 | 4 | 267.49 |
| Ψ(K+M+C+W)p(.) | 1.31 | 0.067 | 6 | 263.41 |
| Ψ(K+W+V)p(.) | 1.57 | 0.061 | 5 | 266.00 |
| Ψ(K+C+V+W)p(.) | 1.73 | 0.056 | 6 | 263.83 |
| Ψ(K+M+C+V+W)p(.) | 2.02 | 0.049 | 7 | 261.73 |
| Ψ(K+W+M)p(.) | 2.33 | 0.042 | 5 | 266.76 |
| Ψ(.)p(.) | 9.58 | 0.001 | 2 | 280.65 |

Covariates considered include; distance to KNP (K), mopane shrubveld (M), combretum/mopane rugged veld (C), distance to settlements (V), distance to water (W), Lebombo hills (L) and sand plains (S). Ψ(.) assumes site use is constant, ∆AICc is the difference in AICc values between each model with the low AICc model, *w* is the AICc model weight, K is the number of parameters in the model, and −2l is twice the negative log-likelihood value.

**Table S5**. Summary of model selection procedure for factors influencing bushmeat poaching site use (Ψ) across 82 sites in the Limpopo National Park, Mozambique.

| **Models** | **∆AICc** | ***w*** | **K** | ***-*2l** |
| --- | --- | --- | --- | --- |
| Ψ(B+A+V)p(T) | 0.00 | 0.648 | 6 | 244.34 |
| Ψ(B+A)p(T) | 2.93 | 0.150 | 5 | 249.60 |
| Ψ(B+A+T+V)p(T) | 4.27 | 0.077 | 7 | 246.22 |
| Ψ(B+A+T)p(T) | 4.52 | 0.068 | 6 | 248.86 |
| Ψ(B+A+R)p(T) | 5.05 | 0.052 | 6 | 249.39 |
| Ψ(.)p(T) | 9.33 | 0.006 | 3 | 260.48 |

Covariates considered include; relative bushmeat abundance (A), relative bushmeat biomass (B), proximity to tracks (T), proximity to settlements (V), proximity to water (W), and ranger patrol effort (R). Detectability (p) varies with tracks (T). Ψ(.) assumes occupancy is constant, ∆AICc is the difference in AICc values between each model with the lowest AICc model, *w* is the AICc model weight, K is the number of parameters in the model, and −2l is twice the negative log-likelihood value.
